# Compensatory Interactions between Corneal and Internal Astigmatism despite Lifestyle Changes

**DOI:** 10.3390/children11020154

**Published:** 2024-01-25

**Authors:** Yuanyuan Liang, Byung-Soo Kang, Chea-Su Kee, Tsz-Wing Leung

**Affiliations:** 1 School of Optometry, The Hong Kong Polytechnic University, Hong Kong; 22117026r@connect.polyu.hk (Y.L.); bs.kang@polyu.edu.hk (B.-S.K.); c.kee@polyu.edu.hk (C.-S.K.); 2 Centre for Eye and Vision Research (CEVR), 17W Hong Kong Science Park, Hong Kong; 3 Research Centre for SHARP Vision (RCSV), The Hong Kong Polytechnic University, Hong Kong

**Keywords:** refractive astigmatism, corneal astigmatism, internal astigmatism, near-work time, outdoor time

## Abstract

This study explores whether children’s refractive errors and visual behaviors reverted to pre-COVID-19 levels a year after normal schooling resumed in Hong Kong as well as the impact of corneal and internal astigmatism on refractive astigmatism development. Vision survey data and questionnaire results collected in 2022 (*n* = 119) and 2020 (*n* = 173) were compared. Cross-sectional data showed similar proportions of astigmatism (cylindrical power ≥ 0.75 D) in the 2020 (49.1%) and 2022 cohorts (55.5%). Despite a 0.28 D increase in corneal astigmatism, a compensatory 0.24 D increase in internal astigmatism of opposite direction kept refractive astigmatism relatively stable. The questionnaire data showed that children spent an additional 0.5 h/day outdoors on weekends post-resumption of normal schooling but engaged in more near-work activities, especially non-screen near-work, by approximately 1 h/day on both weekdays and weekends. These findings were supported by longitudinal data from 72 children who participated in both surveys. This study highlights the significant role of corneal and internal astigmatism in refractive astigmatism changes. Despite the return to in-person classes, children’s total near-work time increased and astigmatism remained high. These findings underscore the need for comprehensive strategies to reduce the high environmental risks for refractive error development in children.

## 1. Introduction

Since the COVID-19 pandemic began in 2020, children’s daily routines have dramatically changed [1]. With lockdowns and the shift to online learning, children are spending more time on digital devices like computers, smartphones, and tablets and less time outside. This change in behavior has been linked to an increase in myopia [2,3,4,5,6,7]. Although extensive research has been undertaken on the pandemic’s impact on myopia, there has been less focus on refractive astigmatism, a common vision condition in children, especially in Asian populations [8,9,10]. Only a few studies have explored whether the pandemic has led to more cases of refractive astigmatism or made existing cases worse [11,12,13].

Refractive astigmatism, unlike myopia, which can be mitigated by reducing viewing distances, blurs the retinal image at all distances by directing a point of light into two perpendicular image foci on separate planes [14]. If left uncorrected, it can influence visual development in children [14,15,16,17]. It was expected that the return to pre-COVID-19 lifestyles following the relaxation of pandemic prevention policies [18,19] would reduce the environmental risk factors for myopia and refractive astigmatism, thereby slowing their development. However, despite the lifting of anti-pandemic measures, myopia prevalence has remained high in mainland China and Hong Kong [20,21]. Conversely, one study reported a return to pre-pandemic levels of refractive astigmatism prevalence in preschool children aged 1 to 4 years after epidemic controls were lifted [12]. To better understand if children’s refractive error and visual behaviors would revert to pre-COVID-19 levels after local pandemic restrictions were eased, we conducted vision screening at a Hong Kong primary school following the resumption of regular in-person classes.

Understanding the origins of increased refractive astigmatism, whether from the cornea or internal optics of the eye, is a critical, yet unaddressed, question in previous studies [11,12,13]. The potential compensatory role of internal astigmatism for corneal astigmatism also remains largely unexplored [22,23,24,25,26]. This highlights the importance of a comprehensive investigation into the origins and progression of refractive astigmatism to fill this significant research gap.

In this follow-up study, we compared data from the COVID-19 home confinement period [11] with post-confinement data. Beyond cross-sectional analysis, we assessed longitudinal changes in ocular parameters and visual behaviors in children who participated in both screenings. Importantly, we analyzed how the cornea or internal optics influenced refractive astigmatism changes and evaluated the compensatory role of internal astigmatism on corneal astigmatism. 

## 2. Methods

### 2.1. Participants

In July 2022, vision screening was performed on Grade 1–6 students at a Hong Kong primary school in the city center. Parents provided informed written consent following a detailed explanation of the study’s purpose. The study adhered to the Declaration of Helsinki and received approval from The Hong Kong Polytechnic University’s ethics committee (HSEARS20220621001).

Out of 397 invited children, 289 participated in the vision screening (72.8% participation rate) and 246 completed the questionnaires. For consistency with our previous data, only children aged 8–10 years (*n* = 143) were included, excluding 103 participants outside this age range. Following the same criteria as the previous study [11], only Chinese children without prior myopia control treatments were included. After excluding 24 non-Chinese children, 119 students were included for analysis. The demographic, refractive error components, and visual behaviors of the 103 excluded Chinese children are detailed in Supplementary Tables S1–S3.

### 2.2. Vision Screening Procedures

Vision screening was conducted during school hours (9 am–1 pm) using the same procedures and instruments as our previous study. Monocular distance visual acuity was measured using the Early Treatment Diabetic Retinopathy Study acuity chart (Precision Vision, La Salle, IL, USA) placed at a distance of 4 m. Non-cycloplegic refraction and keratometry were conducted using an open-field auto-refractometer (Shin-Nippon, NVision-K 5000, Kobe, Japan), where participants were instructed to fixate on a Maltese cross located 6 m away from their eyes; the average was taken from five consecutive readings for each eye. Axial length measurements were obtained using an IOL Master (Carl Zeiss Meditec, Jena, Germany) and, for the analysis, the mean value derived from five consecutive measurements, each with a signal-to-noise ratio exceeding 2.0, was utilized. Prior to each use, both the open-field auto-refractometer and the IOL Master underwent daily calibration.

A thoroughly validated questionnaire was disseminated to parents through schoolteachers to gather essential data on children’s demographics, family history of myopia, and visual behavioral patterns [11]. The visual behaviors examined included the duration spent on diverse activities during non-school hours in the preceding month such as non-screen near-work time, handheld digital screen time, and outdoor time.

It is important to note that non-screen near-work pertains to near-tasks involving all printed materials such as reading, writing, and drawing. In contrast, handheld digital screen time represents the cumulative duration spent on tablets and smartphones. These factors are considered potential risk factors for myopia development and their careful evaluation can provide valuable insights into the prevention and management of refractive errors in children.

### 2.3. Data Analysis

Statistical analyses were conducted using SPSS (version 22, IBM Corp., Armonk, NY, USA) at a significance level of α < 0.05. Right-eye data were used due to the high correlation with left-eye data (Pearson’s correlations; all r ≥ +0.90 and all *p* < 0.001). Refractive astigmatism (RA) was defined as a cylinder power ≥ 0.75 D and classified into three subgroups based on the negative cylindrical axis: with-the-rule (WTR; axis: 0–30° or 150–180°), against-the-rule (ATR; axis: 60–120°), and oblique astigmatism (OBL; axis: 30–60° or 120–150°). Refractive errors were converted into spherical-equivalent refractive errors (SER). RA was decomposed to J_0_ and J_45_ astigmatic components using a Fourier analysis [27]. Positive and negative J_0_ values indicated WTR and ATR astigmatism, respectively; positive and negative J_45_ values indicated oblique astigmatism at 45° and 135°, respectively.

Corneal astigmatism (CA) was calculated as the difference between the flattest and steepest meridian power of the corneal surface as follows:

$$CA=(1.3375-1)\times \left(\frac{1}{r_{f}}-\frac{1}{r_{s}}\right)\times 1000$$

where *r_f_* and *r_s_* refer to the flattest and steepest anterior corneal radii of curvature and 1.3375 is the calibrated refractive index value after considering the posterior cornea contribution [28]. 

Internal astigmatism (IA) was calculated by first adjusting the refractive errors along each principal power meridian to the corneal plane, considering a back vertex distance of 12 mm. This was followed by determining the vector difference between the refractive and corneal J_0_ and J_45_ astigmatic components. Finally, IA was converted into cylindrical power based on these vector components [27]:

$${IA}_{J0}={RA}_{J0}-{CA}_{J0}$$

$${IA}_{J45}={RA}_{J45}-{CA}_{J45}$$

$$IA=-2\sqrt{{{(IA}_{J0})}^{2}+{{(IA}_{J45})}^{2}}$$

Data were presented as mean (±SD) or median (IQR) based on data normality and were verified using the Shapiro–Wilk test. The study compared current data with 2020 data from the same local school to determine the impact of resuming in-person classes on children’s visual behaviors and refractive errors. Continuous variables for each age group were compared using an unpaired *t*-test or the Mann–Whitney U test, while categorical variables were compared using a chi-squared test. Longitudinal data were tested using a paired *t*-test or Wilcoxon signed-rank test.

## 3. Results

### 3.1. Demographic Overview

Table 1 presents the demographic details of the children involved in the 2020 and 2022 vision surveys. The two cohorts exhibited no significant age difference (2020: 9.19 ± 0.81 years vs. 2022: 9.19 ± 0.85 years, unpaired *t*-test, and *p* = 0.98) and a similar gender distribution (55.5% boys in 2020 vs. 52.1% in 2022, chi-squared test, and *p* = 0.57). Parental myopia and family income also showed no significant disparities between the cohorts (chi-squared test; *p* ≥ 0.05).

Upon an age-group comparison, the cohorts showed no significant differences in gender distribution, parental myopia, and family income (chi-squared test; all *p* ≥ 0.20), except for the 10-year-olds, who had higher family incomes in 2022 than in 2020 (chi-squared test, χ^2^ = 6.26, and *p* = 0.012). This highlighted the demographic similarity of the two cohorts, reinforcing the validity of the comparative analysis.

### 3.2. Cross-Sectional Data

#### 3.2.1. Refractive Errors and Axial Length Overview

Table 2 illustrates that the proportions of refractive astigmatism showed no significant differences between the two cohorts (chi-squared test; *p* = 0.29). Similarly, SER (Mann–Whitney test, U = 9668.5, and *p* = 0.38), axial length (unpaired *t*-test, *t* = 0.85, and *p* = 0.40), RA (U = 9532.0; *p* = 0.28), and RA_J0_ (U = 9537.0; *p* = 0.29) components also exhibited no significant disparities. However, the 2022 cohort had a more negative RA_J45_ component than the 2020 cohort (U = 6469.5; *p* < 0.001).

#### 3.2.2. Astigmatism Component Interaction

**Figure 1:**
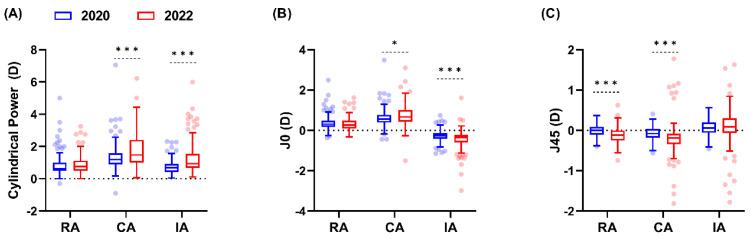
Comparative analysis of astigmatic components between the two cohorts. (**A**) Cylindrical power, (**B**) J_0_, and (**C**) J_45_ astigmatic components for the 2020 (blue) and 2022 (red) cohorts are plotted against refractive (RA), corneal (CA), and internal astigmatic (IA) components. The solid line within the box represents the median, while the box margins denote the interquartile range. The dots represent the outliers from the individual data. Mann–Whitney U test: * *p* < 0.05; *** *p* < 0.001.

In contrast, the 2022 cohort showed significant differences in cylindrical power and the J_0_ and J_45_ components of corneal and internal astigmatism compared with the 2020 cohort (Mann–Whitney test, U ≥ 6363.0, and *p* ≤ 0.017). On average, CA and IA were 0.28 D and 0.24 D higher in 2022 than in 2020 (Figure 1A; U = 7459.0 and 6808.0, respectively; *p* ≤ 0.001). The 2022 cohort exhibited more positive CA_J0_ and more negative CA_J45_ values (Figure 1B,C; U = 8608.0 and 6363.0, respectively; *p* ≤ 0.017). The increase in CA_J0_ was offset by a more negative IA_J0_ (Figure 1B; U = 7616.0; *p* < 0.001), potentially explaining the insignificant change in RA_J0_. No significant difference was found in IA_J45_ between the 2022 and 2020 cohorts (Figure 1C; U = 9586.0; *p* = 0.32).

#### 3.2.3. Astigmatic Subtype Comparison

**Figure 2:**
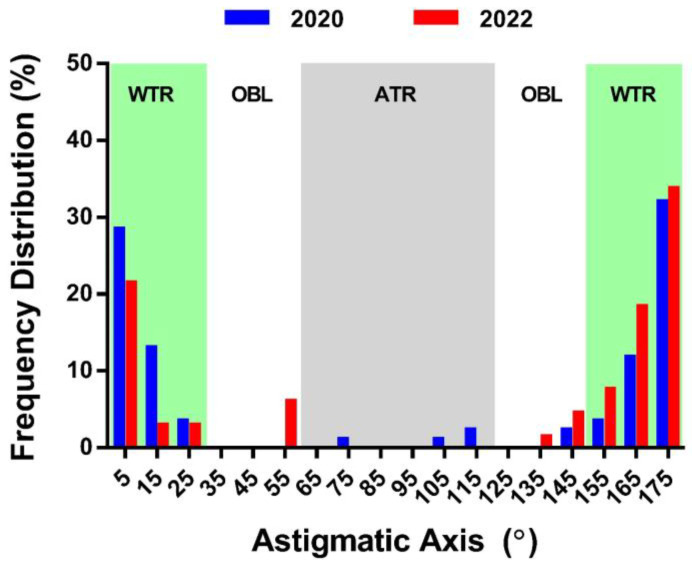
Distribution of astigmatic axis in the right eye. The figure illustrates the proportions of refractive astigmatism per 10° bin width for the 2020 (blue bars) and 2022 (red bars) cohorts. The green, gray, and white areas represent with-the-rule (WTR; axis: 0–30° or 150–180°), against-the-rule (ATR; axis: 60–120°), and oblique (OBL; axis: 30–60° or 120–150°) refractive astigmatism, respectively.

Figure 2 compares the proportions of astigmatic subtypes between the cohorts (Supplementary Figure S1 for left eyes). Among the astigmatic children in the 2022 cohort, WTR refractive astigmatism was the most common subtype (81.8%) followed by OBL (15.2%) and ATR (3.0%). Compared with 2020 data, the 2022 cohort had a higher proportion of OBL refractive astigmatism (chi-squared test, χ^2^ = 10.74, and *p* < 0.001) and a lower proportion of WTR refractive astigmatism (χ^2^ = 11.07; *p* < 0.001). However, astigmatic subtype proportions for the left eyes were similar between the cohorts (see Supplementary Figure S1).

#### 3.2.4. Characteristics of Astigmatism across Three Age Groups

When comparing the two cohorts across three age groups, there were no significant differences in SER, axial length, refractive astigmatism proportion, or RA (with all *p*-values being greater than 0.10). However, the 10-year-old group in 2022 showed a decrease in the RA_J0_ astigmatic component compared with 2020 (U = 1671.5; *p* = 0.036) and the RA_J45_ astigmatism in the 2022 cohort shifted to more negative values across all age groups (U ≥ 277.5; all *p* ≤ 0.013).

In the 2022 cohort, CA significantly increased in the 8-year-old and 9-year-old groups compared with the 2020 cohort (U = 461.0 and 499.0; *p* ≤ 0.009). However, this increase was not observed in the 10-year-old group (U = 1821.0; *p* = 0.16). When breaking down corneal astigmatism into vector components, the 9-year-old group in 2022 had a significantly more positive CA_J0_ than in 2020 (U = 536.0; *p* = 0.011). No significant differences were found in the other two age groups (U ≤ 2104.0; *p* ≥ 0.11). However, CA_J45_ became more negative across all age groups (U ≥ 332.0; all *p* ≤ 0.006), mirroring the changes seen in RAJ_45_.

IA was significantly higher in 2022 than in 2020 across all age groups (U ≥ 432; all *p* ≤ 0.012), with IA_J0_ shifting to more negative values (U ≥ 520.0; all *p* ≤ 0.047). No significant differences were found in IA_J45_ between the two cohorts across all three age groups (U ≤ 1985.0; all *p* ≥ 0.51).

#### 3.2.5. Near-Work and Outdoor Time

In 2022, children increased their near-work activities (i.e., sum of non-screen near-work and digital screen time) on weekdays (Table 3; U = 8504.0, *p* = 0.011, and 1 h/day more) and weekends (U = 7670.5, *p* < 0.001, and 1.5 h/day more) compared with 2020. The 9-year-old and 10-year-old groups spent more time on near-work on weekends in 2022 than in 2020 (U = 554.5 and 1584.0; *p* ≤ 0.01), but the 8-year-old group’s increase was not statistically significant (U = 529.0; *p* = 0.09). No significant differences were found in total near-work time on weekdays among the three age groups (U ≤ 1847.0; *p* ≥ 0.06).

The increase in near-work time in 2022 was due to more non-screen near-work time (U = 7578.0; *p* = 0.045). The same trend was observed on weekends, but it was not statistically significant (U = 6823.5; *p* = 0.066). Only the 8-year-old group showed a significant increase in non-screen near-work time (U = 436.5; *p* = 0.02).

Despite resuming in-person classes, children’s digital screen time (i.e., tablets and smartphones) did not decrease, with no significant differences between the two cohorts (U ≤ 10,234.0; all *p* ≥ 0.10). 

Outdoor time during weekends increased by 0.5 h/day in 2022 (U = 8030.5; *p* = 0.001) but no significant difference was found on weekdays (U = 9081.5; *p* = 0.08). The significant increase was only in the 8-year-old group, both on weekdays and weekends (U = 476.0; *p* = 0.001 and U = 399.5; *p* = 0.001). The other two age groups showed no significant differences in outdoor time between 2020 and 2022 (U ≤ 2095.5; *p* ≥ 0.15). 

### 3.3. Longitudinal Data

#### 3.3.1. Refractive Errors and Axial Length

Of the 72 students who attended vision screenings in both 2020 and 2022, no significant change was observed in RA and RA_J0_ astigmatism (Table 4; Wilcoxon signed-rank test, Z = −1.51 and −0.33, and both *p* ≥ 0.13). However, RA_J45_ astigmatism became more negative in 2022 (Z = −3.67; *p* < 0.001), similar to the changes observed in the cross-sectional data.

Children’s CA significantly increased by 0.28 D (Wilcoxon signed-rank test, Z = −3.74, and *p* < 0.001), as did IA by 0.25 D (Z = −3.23; *p* = 0.001). The compensatory action of the internal optics was evident for the J_0_ astigmatic component, with a +0.06 D shift of CA_J0_ (Z = −1.84; *p* = 0.067) counterbalanced by a −0.07 D shift of IA_J0_ (Z = −2.44; *p* = 0.015). CA_J45_ became more negative with age (Z = −4.33; *p* < 0.001) but the change in IA_J45_ was not significant (Z = −0.44; *p* = 0.66).

Myopia and axial length both significantly increased, by −0.59 D (IQR: −1.12 D and −0.02 D, Wilcoxon signed-rank test, Z = −5.84, and *p* < 0.001) and 0.65 ± 0.41 mm (paired *t*-test, *t* = −13.45, and *p* < 0.001), respectively. 

#### 3.3.2. Near-Work and Outdoor Time

Table 5 presents questionnaire data from children surveyed in both 2020 and 2022. Over two years, total near-work time significantly increased by 1.5 h/day on weekdays (Wilcoxon signed-rank test, Z = −3.53, and *p* < 0.001) and 2 h/day on weekends (Z = −3.52; *p* < 0.001). Non-screen near-work time increased by 1 h/day on both weekdays (Z = −3.90; *p* < 0.001) and weekends (Z = −3.24; *p* = 0.001), and digital screen time increased by 1.25 h/day at weekends (Z = −3.14; *p* = 0.002). Outdoor time increased by 0.5 h/day at weekends (Z = −2.79; *p* = 0.005). Other visual behaviors remained unchanged (all *p* ≥ 0.27).

## 4. Discussion

COVID-19 school suspensions and the shift to online learning increased children’s digital device usage [19,29,30] and decreased their outdoor time [29], which is thought to have contributed to a rise in myopia and refractive astigmatism [11,13]. Despite these alarming changes, our study found that Hong Kong children’s total near-work time, particularly non-screen near-work, increased over the past two years. Although outdoor activities increased after the resumption of in-person classes, the proportions of refractive astigmatism and myopia remained consistent with 2020 levels. Similarly, there were no significant reductions in cylinder power, axial length, and SER.

The primary refractive error change was in the astigmatic axis, with a significant decrease in WTR refractive astigmatism and an increase in oblique refractive astigmatism. This shift was also reflected in refractive J_45_ astigmatism, which displayed a modest, yet statistically significant, negative shift. Interestingly, an analysis of corneal and internal astigmatic changes revealed a notable trend. Despite an average increase in corneal astigmatism, refractive astigmatism remained stable, likely due to a compensatory action from internal optics. Specifically, the J_0_ component of internal astigmatism became more negative to counterbalance the positive shift of corneal J_0_ astigmatism (Figure 1B,C). However, this compensatory mechanism was not evident in the J_45_ astigmatic component. The mechanism behind the interaction between corneal and internal astigmatism is still unclear. However, laboratory studies using human and animal models suggest that the eye can detect and compensate for perceived astigmatic blur, particularly when astigmatism is oriented in WTR and ATR directions [31,32,33,34]. These findings suggest a bias towards the cardinal orientations in the development of refractive astigmatism and warrant further investigation.

Unlike the high astigmatism observed in our study, a recent Chinese study found that the increased prevalence of refractive astigmatism during the COVID-19 pandemic returned to pre-pandemic levels after anti-pandemic measures were relaxed [12]. This study focused on a younger cohort (1–4 years), whose eyes had a high degree of plasticity for growth [35]. Considering the increased near-work time as a contributing factor to the surge in refractive astigmatism in school-aged children [11], it is plausible that this environmental risk factor has less impact on preschool children due to their typically lower exposure to near-work demands. However, the previously mentioned study did not gather data on children’s visual habits [12].

Our study found changes in J_45_ in both refractive and corneal astigmatism between the 2020 and 2022 cohorts, as shown in cross-sectional (Table 2) and longitudinal data (Table 4). This may have been due to an increase in near-work activities in 2022 (Table 3 and Table 5). Screen time on handheld digital devices remained similar to pre-COVID-19 levels on weekdays [11] but increased 1.5-fold on weekends (2018, 2020, and 2022 = 2.0, 2.5, and 3.0 h per day), supporting Wong et al.’s prediction of continued digital device dependence post-COVID-19 [1]. Changes in eyelid pressure from downgaze eye movements during near-work could temporarily reshape the cornea, affecting corneal and refractive astigmatism [36]. Although Shaw et al. [37] reported a change in corneal J_45_ astigmatism after 15 min of reading, other studies found changes in J_0_ astigmatism from smartphone use [38]. However, eyelid-induced corneal distortions can quickly recover post-near-task [39]. Further investigations are needed to determine if these transient corneal shape changes have long-term effects on refractive error development. Moreover, other factors such as ocular rubbing and sleeping habits [40], which may also affect corneal morphology, should be taken into account in future studies to fully understand their potential impact on astigmatism.

This study compared refractive errors and visual behaviors in a Hong Kong primary school over two years, during which the curriculum remained consistent, except for the addition of e-learning during school closures. The study’s strength lies in its use of both cross-sectional and longitudinal data, emphasizing the need to monitor children’s eye health post-pandemic. Limitations include the lack of cycloplegic agents for auto-refraction, potential recall bias from using a questionnaire to collect activity data, and a small sample size from a single city-center school, limiting generalizability. Given that the difference in refractive J_0_ and J_45_ astigmatism in children (aged 6–11 years) measured with or without cycloplegia was negligible (J_0_: −0.08 ± 0.13 D; J_45_: −0.01 ± 0.09 D), the lack of cycloplegia should not have had an impact on the interpretation of astigmatism changes [41]. Additionally, only age-matched children (8–10 years old) were included, excluding 103 children outside this range. Despite these limitations, the consistent results and supportive evidence from the axial length data for myopia development underscored the study’s validity. Future studies could benefit from objective devices [42,43] to quantify near-work behaviors and outdoor time, and should consider high refractive astigmatism in younger children and excessive near-work behaviors in older children.

## 5. Conclusions

In conclusion, post-COVID-19 vision surveys in a primary school revealed that refractive astigmatism, axial length, and myopia in 8- to 10-year-old Hong Kong children remained elevated compared with pre-pandemic levels. Despite the resumption of in-person teaching, children’s near-work time (a potential risk factor for myopia and refractive astigmatism) increased. Our study offers novel insights into the interaction between corneal and internal astigmatism and potential environmental influences on refractive astigmatism changes. Therefore, it is imperative to focus collective efforts on managing children’s visual behaviors to mitigate environmental risk factors for refractive errors. Long-term studies are also crucial to ascertain the enduring impact of the pandemic on children’s ocular health.

## Figures and Tables

**Table 1:** Demographic information (±95% confidence intervals) of participants of 2020 and 2022 cohorts in each age group.

	Total			8-Year-Old			9-Year-Old			10-Year-Old		
	2020	2022	*p*-Value	2020	2022	*p*-Value	2020	2022	*p*-Value	2020	2022	*p*-Value
Sample size	173	119		43	33		54	30		76	56	
Boys (%)	55.5 (48.0, 63.0)	52.1 (43.0, 61.2)	0.57	55.8 (40.3, 71.3)	45.5 (27.5, 63.4)	0.37	51.9 (38.1, 65.6)	63.3 (45.0, 81.6)	0.31	57.9 (46.5, 69.3)	50.0 (36.5, 63.5)	0.37
Monthly family income (%)			0.05			0.31			0.21			**0.012**
≤HKD 19,999	59.5 (52.2, 66.9)	47.9 (38.8, 57.0)		51.2 (35.6, 66.7)	39.4 (21.8, 57.0)		70.4 (57.8, 83.0)	56.7 (37.8, 75.5)		69.7 (59.2, 80.3)	48.2 (34.7, 61.7)	
>HKD 19,999	40.5 (33.1, 47.8)	52.1 (43.0, 61.2)		48.8 (33.3, 64.4)	60.6 (43.0, 78.2)		29.6 (17.0, 42.2)	43.3 (24.5, 62.2)		30.3 (19.7, 40.8)	51.8 (38.3, 65.3)	
Parental Myopia (%) ^#^	57.2 (49.8, 64.7)	63.9 (55.1, 72.6)	0.26	81.4 (69.3, 93.5)	69.7 (53.1, 86.2)	0.23	53.7 (40.0, 67.4)	63.3 (45.0, 81.6)	0.39	46.7 (35.1, 58.2)	60.7 (47.5, 73.9)	0.20

Note. HKD: Hong Kong Dollar. ^#^ At least one parent with myopia. Figures in bold indicate statistical significance. All comparisons were performed using chi-squared tests.

**Table 2:** Results of astigmatic components (median (IQR)) and axial length (mean ± SD) for the cross-sectional survey data.

	Total			8-Year-Old			9-Year-Old			10-Year-Old		
	2020	2022	*p*-Value	2020	2022	*p*-Value	2020	2022	*p*-Value	2020	2022	*p*-Value
Astigmatism proportion (%)	49.1 (41.6, 56.7)	55.5 (46.4, 64.5)	0.29	44.2 (28.7, 59.7)	57.6 (39.8, 75.4)	0.25	42.6 (29.0, 56.2)	46.7 (27.7, 65.6)	0.72	56.6 (45.2, 68.0)	58.9 (45.6, 72.2)	0.79
RA (D) *	0.62 (0.50, 1.00)	0.75 (0.50, 1.12)	0.28	0.62 (0.37, 0.87)	0.87 (0.56, 1.06)	0.10	0.62 (0.50, 0.87)	0.62 (0.50, 1.15)	0.25	0.75 (0.53, 1.09)	0.75 (0.37, 1.09)	0.57
RA_J0_ (D) *	0.31 (0.18, 0.45)	0.26 (0.10, 0.49)	0.29	0.25 (0.17, 0.42)	0.29 (0.14, 0.53)	0.69	0.28 (0.18, 0.40)	0.25 (0.17, 0.55)	0.78	0.36 (0.17, 0.53)	0.28 (0.04, 0.48)	**0.036**
RA_J45_ (D) *	0 (−0.10, 0.09)	−0.12 (−0.25, 0)	**<0.001**	0.03 (−0.04, 0.15)	−0.14 (−0.01, 0.28)	**<0.001**	−0.01 (−0.09, 0.09)	−0.12 (−0.20, 0.01)	**0.008**	−0.03 (−0.14, 0.06)	−0.12 (−0.24, 0)	**0.013**
CA (D)	1.19 (0.89, 1.58)	1.47 (0.99, 2.41)	**<0.001**	1.10 (0.84, 1.34)	1.57 (0.93, 2.42)	**0.009**	1.13 (0.71, 1.46)	1.53 (0.98, 2.69)	**0.004**	1.33 (0.91, 1.82)	1.43 (1.02, 2.12)	0.16
CA_J0_ (D)	0.57 (0.39, 0.77)	0.67 (0.43, 1.00)	**0.017**	0.53 (0.38, 0.65)	0.69 (0.41, 1.00)	0.11	0.54 (0.34, 0.72)	0.71 (0.44, 1.22)	**0.011**	0.65 (0.43, 0.89)	0.60 (0.43, 0.89)	0.91
CA_J45_ (D)	−0.08 (−0.18, 0.04)	−0.19 (−0.34, 0.08)	**<0.001**	−0.05 (−0.14, 0.04)	−0.20 (−0.34, −0.08)	**<0.001**	−0.08 (−0.16, 0.04)	−0.15 (−0.41, −0.06)	**0.004**	−0.09 (−0.20, 0.05)	−0.20 (−0.33, −0.09)	**0.006**
IA (D)	0.69 (0.42, 0.91)	0.93 (0.66, 1.55)	**<0.001**	0.68 (0.40, 0.88)	0.94 (0.66, 1.46)	**0.004**	0.70 (0.40, 0.90)	0.81 (0.66, 1.84)	**0.012**	0.75 (0.50, 1.01)	0.97 (0.67, 1.52)	**0.003**
IA_J0_ (D)	−0.25 (−0.41, −0.13)	−0.35 (−0.61, −0.22)	**<0.001**	−0.25 (−0.37, −0.11)	−0.36 (−0.53, −0.22)	**0.047**	−0.26 (−0.39, −0.13)	−0.34 (−0.85, −0.16)	**0.042**	−0.26 (−0.44, −0.14)	−0.34 (−0.62, −0.23)	**0.016**
IA_J45_ (D)	0.06 (−0.05, 0.20)	0.08 (−0.06, 0.28)	0.32	0.09 (−0.02, 0.21)	0.08 (−0.05, 0.34)	0.75	0.03 (−0.09, 0.20)	0.07 (−0.10, 0.21)	0.59	0.07 (−0.08, 0.18)	0.09 (−0.10, 0.33)	0.51
SER (D)	−1.38 (−2.22, −1.00)	−1.50 (−2.75, −0.94)	0.38	−1.19 (−1.81, −0.93)	−1.50 (−2.85, −0.91)	0.15	−1.47 (−2.21, −1.06)	−1.56 (−2.83, −1.09)	0.61	−1.53 (−2.53, −1.00)	−1.47 (−2.75, −0.93)	0.89
Axial length (mm)	23.67 ± 0.95	23.77 ± 1.08	0.40	23.47 ± 0.94	23.37 ± 1.12	0.66	23.62 ± 0.84	23.87 ± 1.05	0.23	23.81 ± 1.02	23.95 ± 1.03	0.45

* For comparison with other epidemiological studies, refractive astigmatism and its vector components were presented on the spectacle plane. Figures in bold indicate statistical significance. Except for axial length, which was compared using an unpaired *t*-test, all pairwise comparisons were performed using Mann–Whitney U tests.

**Table 3:** Time spent on various visual activities (median (IQR)) for the cross-sectional survey data.

	Total			8-Year-Old			9-Year-Old			10-Year-Old		
	2020	2022	*p*-Value	2020	2022	*p*-Value	2020	2022	*p*-Value	2020	2022	*p*-Value
Total near-work time (h/day)												
Weekdays	3.50 (2.50, 5.00)	4.50 (2.00, 6.50)	**0.011**	3.00 (2.00, 5.00)	5.00 (2.00, 7.50)	0.058	4.00 (3.00, 5.00)	4.50 (2.88, 7.13)	0.16	3.00 (2.00, 5.00)	4.00 (1.78, 6.00)	0.19
Weekends	4.00 (3.00, 5.25)	5.50 (3.00, 8.00)	**<0.001**	4.00 (3.00, 6.00)	6.00 (2.25, 9.00)	0.087	4.00 (3.00, 5.50)	5.50 (3.88, 8.88)	**0.016**	4.00 (2.13, 5.00)	5.25 (3.00, 7.75)	**0.012**
Non-screen time (h/day)												
Weekdays	1.00 (1.00, 2.00)	2.00 (1.00, 3.00)	**0.045**	1.00 (1.00, 2.00)	2.00 (1.00, 3.00)	**0.017**	1.50 (1.00, 2.00)	2.00 (1.00, 3.00)	0.35	1.00 (0.50, 2.00)	2.00 (0.50, 3.75)	0.35
Weekends	1.25 (1.00, 2.00)	2.00 (1.00, 3.00)	0.066	1.00 (1.00, 2.00)	2.00 (1.00, 4.00)	0.075	1.75 (1.00, 2.00)	2.00 (0.50, 2.63)	0.76	1.00 (0.50, 2.00)	2.00 (1.00, 3.00)	0.12
Screen time (h/day)												
Weekdays	2.00 (1.00, 3.00)	2.00 (1.00, 4.00)	0.93	2.00 (1.00, 4.00)	2.00 (1.00, 5.00)	0.65	2.00 (1.50, 3.00)	2.00 (1.19, 4.00)	0.80	2.00 (1.00, 3.00)	2.00 (1.00, 3.75)	0.58
Weekends	2.50 (2.00, 4.00)	3.00 (1.50, 6.00)	0.11	3.00 (1.50, 5.00)	3.00 (1.00, 6.50)	0.65	2.25 (1.88, 4.00)	3.50 (2.00, 7.25)	0.10	2.25 (1.25, 4.00)	3.00 (1.50, 5.00)	0.39
Outdoor time (h/day)												
Weekdays	1.00 (0, 1.00)	1.00 (0, 2.00)	0.077	0.50 (0, 1.50)	1.50 (0.25, 2.00)	**0.011**	1.00 (0, 1.00)	0.75 (0, 1.50)	0.66	1.00 (0, 1.00)	1.00 (0, 1.88)	0.88
Weekends	1.50 (1.00, 2.00)	2.00 (1.00, 3.50)	**0.001**	1.00 (1.00, 2.00)	2.50 (1.00, 4.25)	**0.001**	2.00 (1.00, 2.00)	2.00 (1.00, 3.00)	0.32	2.00 (0.50, 2.00)	2.00 (0.85, 3.88)	0.15

Figures in bold indicate statistical significance. All pairwise comparisons were performed using Mann–Whitney U tests.

**Table 4:** Longitudinal astigmatic changes in children surveyed in 2020 and 2022 (*n* = 72).

	2020	2022	*p*-Value
RA (D)	0.62 (0.50, 0.87)	0.75 (0.40, 1.12)	0.13
RA_J0_ (D)	0.26 (0.15, 0.38)	0.22 (0.06, 0.50)	0.74
RA_J45_ (D)	0.01 (−0.08, 0.11)	−0.06 (−0.24, 0.02)	**<0.001**
CA (D)	1.16 (0.84, 1.41)	1.44 (0.98, 1.94)	**<0.001**
CA_J0_ (D)	0.56 (0.36, 0.69)	0.62 (0.38, 0.88)	0.067
CA_J45_ (D)	−0.05 (−0.14, 0.07)	−0.20 (−0.33, −0.06)	**0.001**
IA (D)	0.70 (0.45, 0.90)	0.95 (0.64, 1.39)	**0.001**
IA_J0_ (D)	−0.27 (−0.40, −0.14)	−0.35 (−0.54, −0.23)	**0.015**
IA_J45_ (D)	0.03 (−0.08, 0.20)	0.08 (−0.12, 0.30)	0.66
SER (D)	−1.28 (−1.98, −1.00)	−1.94 (−3.00, −1.05)	**<0.001**
Axial length (mm)	23.50 ± 0.89	24.15 ± 1.01	**<0.001**

Figures in bold indicate statistical significance. Except for axial length, which was compared using a paired *t*-test, all other comparisons were performed using the Wilcoxon signed-rank test.

**Table 5:** Longitudinal changes in activity time (median (IQR)) for children surveyed in 2020 and 2022 (*n* = 72).

	2020	2022	*p*-Value
Total near-work time (h/day)			
Weekdays	3.50 (2.63, 5.00)	5.00 (2.50, 7.00)	**<0.001**
Weekends	4.00 (3.00, 5.38)	6.00 (3.63, 9.00)	**<0.001**
Non-screen time (h/day)			
Weekdays	1.00 (0.50, 2.00)	2.00 (1.00, 3.00)	**<0.001**
Weekends	1.00 (0.50, 2.00)	2.00 (1.00, 3.00)	**0.001**
Screen time (h/day)			
Weekdays	2.00 (1.00, 3.38)	2.25 (1.50, 4.00)	0.053
Weekends	2.75 (2.00, 4.00)	4.00 (1.63, 6.00)	**0.002**
Outdoor time (h/day)			
Weekdays	0.5 (0, 1.00)	1.00 (0, 1.50)	0.21
Weekends	1.50 (1.00, 2.00)	2.00 (1.00, 4.00)	**0.005**

Figures in bold indicate statistical significance. All comparisons were performed using the Wilcoxon signed-rank test.

## Data Availability

The data presented in this study are available on request from the corresponding author. The data are not publicly available due to specific ethical and privacy considerations.

## Supplementary Materials

The following supporting information can be downloaded at: https://www.mdpi.com/article/10.3390/children11020154/s1, Table S1. Demographic information (±95% confidence intervals) of excluded Chinese participants. Table S2. Refractive-error components [Median (IQR)] and axial length (Mean ± SD) of excluded Chinese participants. Table S3. Time spent on various visual activities [Median (IQR)] of excluded Chinese participants. Figure S1. Frequency distribution of astigmatic axis of left eyes. Proportions of astigmatism per 10°-bin width for the 2020 (blue bars) and 2022 (red bars) cohorts. In each plot, the green, gray, and white areas represent With-The-Rule (WTR, axis: 0°–30° or 150°–180°), Against-The-Rule (ATR, axis: 60°–120°), and OBLique (OBL, axis: 30°–60° or 120°–150°) astigmatism, respectively. Among the astigmatic children, WTR astigmatism was the main astigmatic subtype (94.5%) in the 2022 cohort, followed by oblique astigmatism (4.1%) and ATR astigmatism (1.4%). Compared to data collected in 2020, the proportion of all three types of astigmatism was similar to children in the 2022 cohort (Chi-squared test, all *p* ≥ 0.49 test).
