# Supplementary material for: Compensatory Interactions between Corneal and Internal Astigmatism despite Lifestyle Changes
Source: Children (Basel). 2024 Jan 25;11(2):154. doi: 10.3390/children11020154 (PMC10887046; doi:10.3390/children11020154)
Supplement: Supplementary file 1 [file children-11-00154-s001.zip › children-2830698-supplementary.pdf]

Table S1. Demographic information (±95% confidence intervals) of excluded Chinese participants.

	**≤7 years old**	**11 years old**	**≥ 12 years old**
Sample Size	37	28	38
Boys (%)	54.1 (37.2, 70.9)	42.9 (23.3, 62.4)	52.6 (36.0, 69.3)
Monthly Family Income (%)			
≤ HK$19,999	48.6 (31.8, 65.5)	53.6 (33.9, 73.3)	57.9 (41.4, 74.3)
> HK$19,999	51.4 (34.5, 68.2)	46.4 (26.7, 66.1)	42.1 (25.7, 58.6)
Parental Myopia (%)[#]	62.2 (45.8, 78.6)	57.1 (37.6, 76.7)	42.1 (25.7, 58.6)

# at least one parent with myopia.

Table S2. Refractive-error components [Median (IQR)] and axial length (Mean±SD) of excluded Chinese participants.

	**≤7 years old**	**11 years old**	**≥ 12 years old**
Astigmatism Proportion (%)	81.1<br>(67.8, 94.3)	50.0<br>(30.3, 69.7)	63.2<br>(47.1, 79.2)
Cylinder Power (D)	0.87<br>(0.75, 1.12)	0.69<br>(0.50, 0.97)	0.81<br>(0.47, 1.12)
J_0 (D)	0.34<br>(0.04, 0.51)	0.20<br>(+0.05, 0.43)	0.29<br>(0.15, 0.42)
J_{45} (D)	–0.08<br>(–0.21, 0.19)	–0.06<br>(–0.22, 0.05)	–0.11<br>(–0.27, 0)
SER (D)	–1.37<br>(–1.88, –0.97)	–2.13<br>(–3.11, –1.14)	–2.19<br>(–3.78, –1.06)
Axial Length (mm)	23.20±0.99	24.22±1.18	24.37±0.92

Table S3. Time spent on various visual activities [Median (IQR)] of excluded Chinese participants.

		≤7 years old	11 years old	≥ 12 years old
Total Near Work Tme (hr/day)				
	Weekdays	3.00	6.00	6.00
		(2.00, 6.00)	(2.75, 7.00)	(4.88, 8.00)
	Weekends	5.00	7.00	8.00
		(3.00, 7.50)	(4.00, 10.75)	(5.38, 12.63)
Non-screen Time (hr/day)				
	Weekdays	1.50	1.50	3.00
		(0.50, 2.00)	(0.50, 3.00)	(1.00, 4.00)
	Weekends	2.00	1.00	2.00
		(1.00, 3.00)	(0.20, 2.75)	(0.50, 3.13)
Screen Time (hr/day)				
	Weekdays	2.00	3.50	3.75
		(0.65, 4.00)	(2.00, 4.75)	(2.00, 4.00)
	Weekends	3.00	6.00	5.75
		(1.00, 5.50)	(3.00, 8.00)	(3.75, 8.25)
Outdoor Time (hr/day)				
	Weekdays	1.50	0.50	1.00
		(0, 3.00)	(0, 1.00)	(0, 3.00)
	Weekends	3.00	2.00	2.50
		(1.00, 5.75)	(0.35, 4.00)	(0, 5.00)

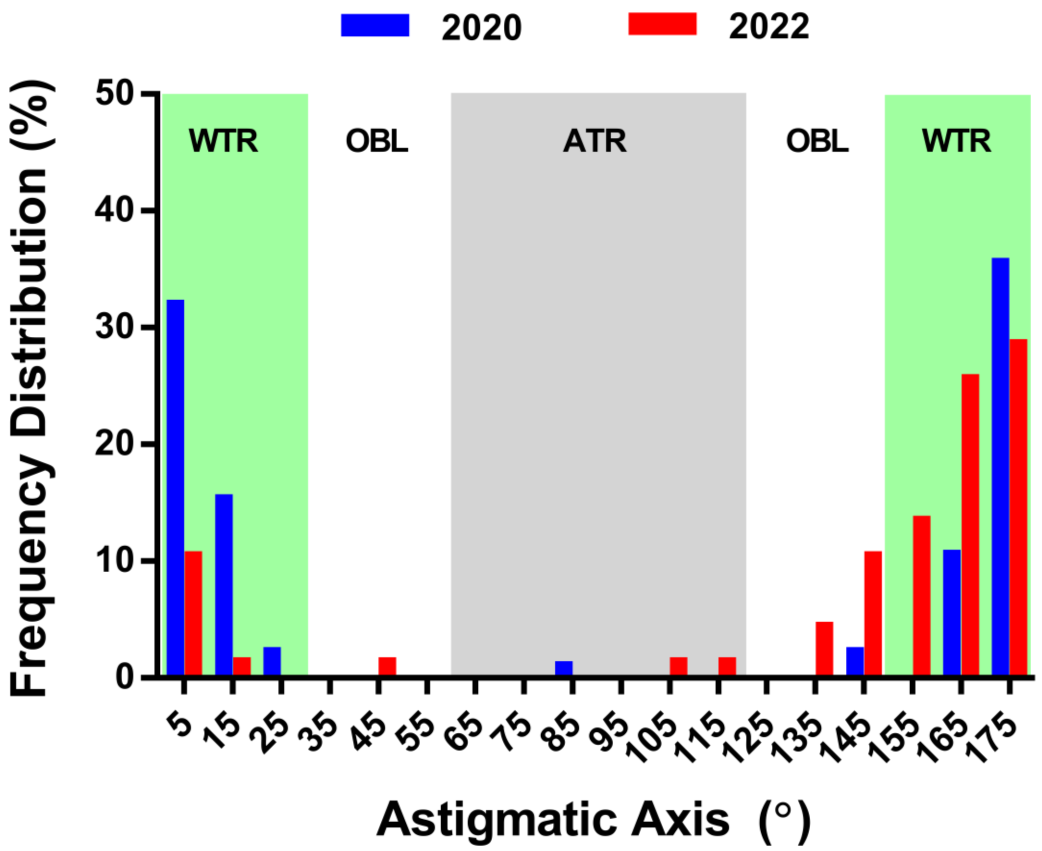

Figure S1. Frequency distribution of astigmatic axis of left eyes. Proportions of astigmatism per 10°-bin width for the 2020 (blue bars) and 2022 (red bars) cohorts. In each plot, the green, gray, and white areas represent With-The-Rule (WTR, axis: 0°-30° or 150°-180°), Against-The-Rule (ATR, axis: 60°-120°), and OBLique (OBL, axis: 30°-60° or 120°-150°) astigmatism, respectively. Among the astigmatic children, WTR astigmatism was the main astigmatic subtype (94.5%) in the 2022 cohort, followed by oblique astigmatism

(4.1%) and ATR astigmatism (1.4%). Compared to data collected in 2020, the proportion of all three types of astigmatism was similar to children in the 2022 cohort (Chi-squared test, all $p \geq 0.49$).
